# Periodontitis leads to VAP in ICU patients: A dental note

**DOI:** 10.4103/0975-7406.72146

**Published:** 2010

**Authors:** Rajiv Saini, Santosh Saini, Sugandha Sharma

**Affiliations:** Department of Periodontology and Oral Implantology, Rural Dental College-Loni, Ahmednagar, Maharashtra, India. E-mail: drperiodontist@yahoo.co.in; 1 Department of Microbiology, Rural Dental College-Loni, Ahmednagar, Maharashtra, India; 2 Department of Prosthodontics, Rural Dental College-Loni, Ahmednagar, Maharashtra, India

Sir,

Periodontitis is a destructive inflammatory disease of the supporting tissues of the teeth and is caused by specific microorganisms or group of specific microorganisms resulting in progressive destruction of periodontal ligament and alveolar bone with periodontal pocket formation, gingival recession, or both.[1,2] The host responds to the periodontal infections with an array of events involving both innate and adaptive immunity. Periodontitis has been proposed as having an etiological or modulating role in cardiovascular and cerebrovascular disease, diabetes, respiratory disease, and adverse pregnancy outcome. Several mechanisms have been proposed to explain or support such theories. Oral lesions are indicators of disease progression and oral cavity can be an window to overall health.[3] Bacteria are the prime etiological agent in periodontal disease, and it is estimated that more than 500 different bacterial species are capable of colonizing the adult mouth.[1] The lesions of the oral cavity have an immense impact on the quality of life of the patient with complex advance diseases.[3] Oral care is an important component of intensive care nursing but is often given low priority when compared with other critical practices. Recent evidence indicates that colonization of the mouth with respiratory pathogens may contribute to the ventilator-associated pneumonia (VAP). Oral care may be an important preventive measure against VAP and not merely a comfort measure.[4] Normal oral flora has been shown to be altered in ICU patients, with the normal aerobic oral organisms being replaced by mainly Gram-negative organisms, and the microaspiration of oropharyngeal secretions is well recognized as a significant risk factor in the development of VAP.[5] Critically ill patients also have impaired immunological deficiencies and may be unable to respond to bacterial invasion of the lungs. Pathogens commonly responsible for the nosocomial pneumonia in ICU patients were found to colonize in the dental plaque and oral mucosa of these patients.[6] Assessment of the oropharynx and maintaining a favorable level of hygiene are difficult tasks to perform in both critically ill and in orally incubated patients due to the lack of access to the oral cavity.[7] The orally incubated patient is at an even greater risk of colonization of organisms because mouth care is often hampered by the presence of tape, tubes, and bite blocks.[8] Various strategies have been suggested to prevent pathological oral colonization including selective digestive tract decontamination with topical antibiotics, application of an antiseptic mouthwash, and oral hygiene packages including tooth-brushing; there is some evidence to suggest that oral care, including tooth-brushing, is more effective at removing plaque than foam swabs alone and electric toothbrushes have been demonstrated to be superior to manual ones in the removal of plaque.[5] Thus, simple measures such as providing adequate oral hygiene may provide a simple and cost-effective method of reducing the incidence of VAP and consequently morbidity and mortality in ICU patients.
